# Interplay Among Metabolism, Epigenetic Modifications, and Gene Expression in Cancer

**DOI:** 10.3389/fcell.2021.793428

**Published:** 2021-12-24

**Authors:** Miaomiao Huo, Jingyao Zhang, Wei Huang, Yan Wang

**Affiliations:** ^1^ Key Laboratory of Cancer and Microbiome, State Key Laboratory of Molecular Oncology, National Cancer Center/National Clinical Research Center for Cancer/Cancer Hospital, Chinese Academy of Medical Sciences and Peking Union Medical College, Beijing, China; ^2^ Beijing Key Laboratory of Cancer Invasion and Metastasis Research, Department of Biochemistry and Molecular Biology, School of Basic Medical Sciences, Capital Medical University, Beijing, China

**Keywords:** epigenetic modifications, metabolic reprogramming, metabolic enzymes, gut microbiota, clinical trails

## Abstract

Epigenetic modifications and metabolism are two fundamental biological processes. During tumorigenesis and cancer development both epigenetic and metabolic alterations occur and are often intertwined together. Epigenetic modifications contribute to metabolic reprogramming by modifying the transcriptional regulation of metabolic enzymes, which is crucial for glucose metabolism, lipid metabolism, and amino acid metabolism. Metabolites provide substrates for epigenetic modifications, including histone modification (methylation, acetylation, and phosphorylation), DNA and RNA methylation and non-coding RNAs. Simultaneously, some metabolites can also serve as substrates for nonhistone post-translational modifications that have an impact on the development of tumors. And metabolic enzymes also regulate epigenetic modifications independent of their metabolites. In addition, metabolites produced by gut microbiota influence host metabolism. Understanding the crosstalk among metabolism, epigenetic modifications, and gene expression in cancer may help researchers explore the mechanisms of carcinogenesis and progression to metastasis, thereby provide strategies for the prevention and therapy of cancer. In this review, we summarize the progress in the understanding of the interactions between cancer metabolism and epigenetics.

## Introduction

Cancer is a disease with high morbidity and mortality and is a serious threat to human health (Bray et al., 2021). Genome instability and mutations contribute to the extraordinary diversity of cancer, and tumors acquire multiple hallmarks during their multistep development, including the reprogramming of energy metabolism (Hanahan and Weinberg, 2011; Sung et al., 2021). Over the past few decades, researchers have found that the metabolic characteristics of tumor cells are significantly different from those of normal cells. Tumor cells have high nutrient and energy requirements, based on their characteristics of sustaining proliferative signaling, evading growth suppressors, and deregulating cellular energetics (Hanahan and Weinberg, 2011). The metabolic reprogramming of tumor cells enables them to obtain essential nutrients from a nutrition-deficient environment to maintain continuous cell growth and proliferation (Pavlova and Thompson, 2016). On one hand, the activation of oncogenes and the deficiency of tumor suppressor genes promote the metabolic reprogramming of tumors, to achieve stronger nutrient-utilization ability and provide material and energy for biosynthesis. At the same time, a lack of nutrition in solid tumors also requires malignant tumor cells to possess metabolic flexibility to maintain growth and survival (Boroughs and DeBerardinis, 2015). Altered metabolic profiles mainly occur with respect to the uptake and metabolism of glucose and amino acids and the synthesis of lipids. For example, the Warburg effect, observed in cancer, suggests that even under sufficient oxygen conditions, malignant tumor cells are active in anaerobic glycolysis, which yields lactic acid, instead of oxidative phosphorylation like that in normal differentiated cells, characterized by a high glucose uptake rate and active glycolysis; moreover, a high level of the metabolite lactic acid correlates with poor tumor prognosis (Warburg, 1956; Som et al., 1980; Vander Heiden et al., 2009). Although aerobic glycolysis is an inefficient way to generate adenosine 5′-triphosphate (ATP), it meets the nutrient needs for cancer cell proliferation via the incorporation of metabolized nutrients into biomass. This reveals a link between cellular metabolism and cell growth control (Vander Heiden et al., 2009). As a highly versatile nutrient, glutamine is also important for tumor cell growth. Cancer cells take up glutamine via glutamine transporter (ASCT2), also known as solute carrier family 1 member 5 (SLC1A5) (Wang et al., 2015). Further, cancer cells are addicted to glutamine through oncogene-dependent pathways involving c-MYC, AKT (Serine/Threonine Kinase 1), and p53 (Tumor Protein P53) (Wettersten et al., 2017). Likewise, as a principal growth-supporting substrate, glutamine provides nitrogen for the biosynthesis of purine and pyrimidine nucleotides, glucosamine-6-phosphate, and nonessential amino acids (Pavlova and Thompson, 2016). Additionally, a process known as glutaminolysis can divert abundant glutamine to replenish the tricarboxylic acid (TCA) cycle (Wong et al., 2017). Lifestyle-related factors, especially diet and nutrition, have a profound effect on human health. Moreover, the gut microbiota plays an important role in this by further metabolizing nutrients from the diet and producing a variety of chromatin-modifying compounds, ultimately regulating histone methylation and acetylation by modulating the intracellular pools of metabolites (Dai et al., 2020).

Heritable changes in gene expression that do not include changes to the DNA sequence itself are termed epigenetic changes. Epigenetics mainly are manifested as DNA methylation, histone post-translational modifications (PTMs), such as acetylation, methylation, phosphorylation, ubiquitination, glycosylation, lactylation, succinylation, and other acyl modifications, including O-linked N-acetylglucosamine modification (O-GlcNAcylation) (Esteller, 2008; Tan et al., 2011; Suva et al., 2013; Huang et al., 2014; Tessarz and Kouzarides, 2014; Dai et al., 2020), as well as RNA methylation, including N6-methyladenosine (m^6^A) and 5-methylcytosine (m^5^C) (Han et al., 2020). Epigenetic plasticity in the process of tumorigenesis and development facilitates the acquisition of hallmark characteristics of cancer (Esteller, 2008; Hanahan and Weinberg, 2011). Many metabolic intermediates serve as epigenetic modification substrates or cofactors, and the various epigenetic processes are primarily governed by the concentrations of the involved reactants (Simithy et al., 2017; Wagner et al., 2017; Thakur and Chen, 2019).

Epigenetic modificatios and metabolism are two fundamental biological processes. Epigenetic alterations and metabolic reprogramming in cancer are highly interrelated. Oncogene-driven metabolic reprogramming alters the epigenetic landscape by regulating DNA and histone modification-related enzyme activity. However, the expression of metabolic genes is regulated by epigenetic mechanisms, thus, altering the metabolome. Therefore, the crosstalk between epigenetics and metabolism plays a crucial role in carcinogenesis and cancer progression through the proliferation, metastasis, and heterogeneity of cancer cells.

## Epigenetic Modifications

Increasing evidence suggests that cancer is a metabolic disease. Epigenetic regulation plays a crucial role in metabolic regulation and tumorigenesis (Li et al., 2018; Bates, 2020). To maintain homeostasis and ensure cell survival, cells must dynamically respond to changes in the environment and reprogram their metabolic state. Similarly, the harmonization of gene expression is required to ensure normal cell function. Epigenetic modifications provide an ideal mechanism for the regulation of gene expression and metabolic reprogramming. Specifically, changes in histone modification are fast and reversible and rely on metabolic intermediates as cofactors for modification. Therefore, it is of great significance to understand the relationship among the metabolic environment, epigenetic modifications, and the expression of genes that play a role in many diseases, particularly cancer (Li et al., 2018).

### Deoxyribonucleic Acid and Ribonucleic Acid Methylation

**FIGURE 1:**
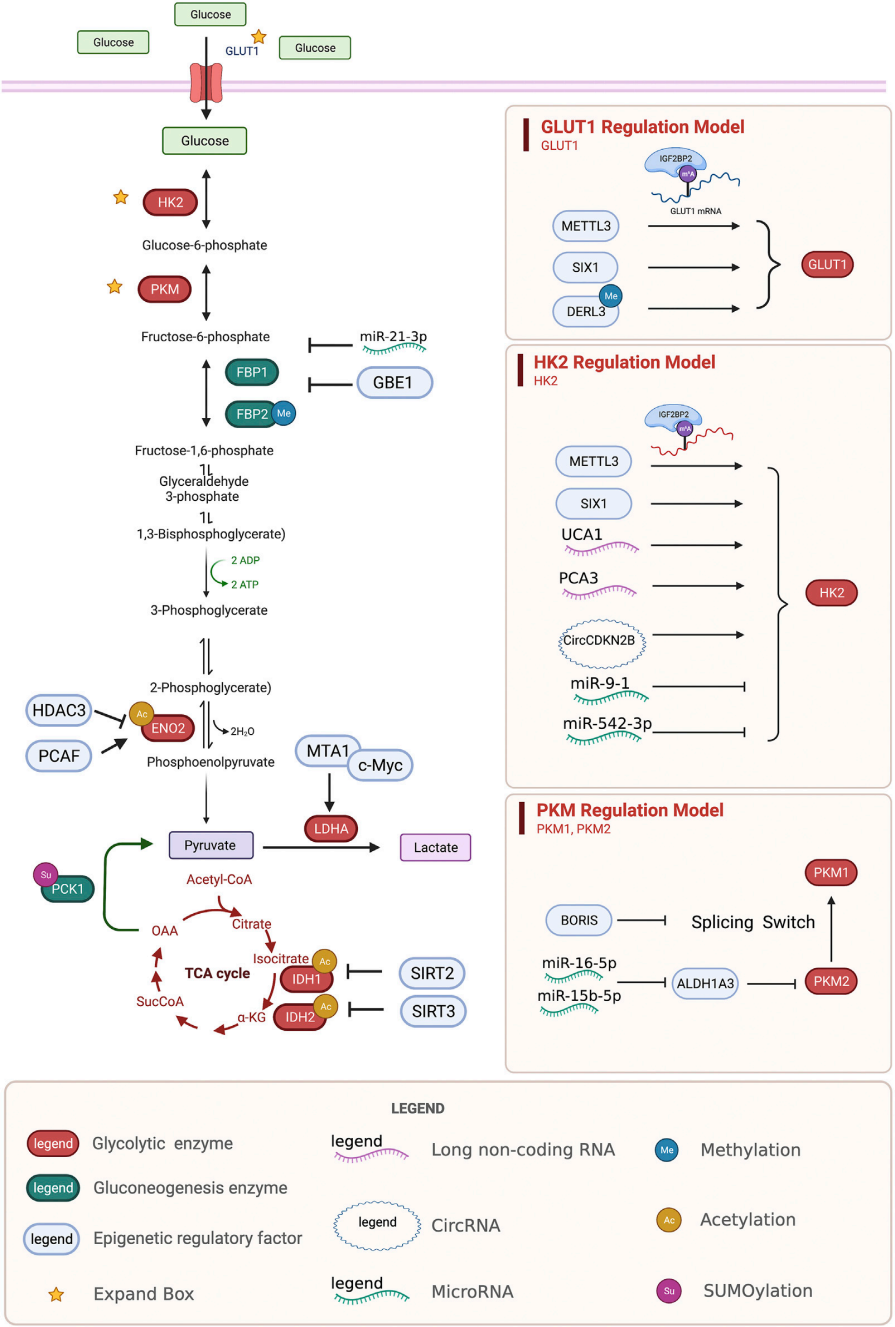
Regulation of glycolysis and gluconeogenesis enzymes by epigenetic modifications. Epigenetic regulatory enzymes, lncRNA, and circRNA regulate the epigenetic regulation of the key proteins, GLUT1, HK2, PKM, ENO2, LDHA, IDH, and the key enzymes FBP and PCK in the process of gluconeogenesis. GLUT1, glucose transporter type 1; HK2, hexokinase 2; PKM, pyruvate kinase M1/2; ENO2, enolase 2; LDHA, lactate dehydrogenase A; IDH, isocitrate dehydrogenase (NADP (+)); FBP, fructose-bisphosphatase; PCK, phosphoenolpyruvate carboxykinase.

Many studies have shown that DNA and RNA methylation plays an important role in regulating cancer metabolism (Saghafinia et al., 2018; Uddin et al., 2021). Abnormal methylation in the promoter region of tumor suppressor genes is key to tumorigenesis and cancer development (Nishiyama and Nakanishi, 2021). Zinc finger DHHC-Type containing 1 (*ZDHHC1*), a recently discovered tumor suppressor gene, is silenced in a variety of cancers through abnormal hypermethylation to inhibit glucose metabolism and the pentose phosphate pathway (Le et al., 2020). The transcription factor brother of the regulator of imprinted sites (BORIS) regulates the methylation of pyruvate kinase M1/2 (*PKM*) exons and the alternative splicing of *PKM* mRNA to mediate the Warburg effect and promote breast cancer (Singh et al., 2017). In colon cancer, hypermethylation of the derlin3 (*DERL3*) promoter region promotes high expression of solute carrier family 2 member 1 (*SLC2A1*) to enhance the Warburg effect (Lopez-Serra et al., 2014). Methyltransferase 3, N6-adenosine-methyltransferase complex catalytic subunit 3 (METTL3) attaches m6A-IGF2BP2/3 to stabilize the expression of hexokinase 2 (HK2), resulting in the expression of phosphorylated glucose hexokinases, and *SLC2A1*, resulting in the expression of glucose transporter (GLUT1), in colon cancer to activate the glycolytic pathway (Shen et al., 2020) (Figure 1).

The reduced transcription of fructose bisphosphates 1 (*FBP1*)*,* a rate-limiting enzyme in gluconeogenesis, mediates disruptions to gluconeogenesis and increased glycolytic activity, causing tumor progression and poor prognosis (Hirata et al., 2016). Under hypoxic conditions, HIF1a promotes the methylation of *FBP1* by upregulating the expression of glycogen-branching enzyme 1 (GBE1) in lung adenocarcinoma (Li L. et al., 2020). The promoter region of fructose-bisphosphatase 2 (*FBP2*) in gastric cancer is densely methylated to promote glucose metabolism (Li et al., 2013) (Figure 1).

Peroxisome proliferator activated receptor alpha (PPARα), a nuclear receptor that regulates lipid homeostasis, inhibits DNA methyltransferase 1 (DNMT1)-mediated cyclin dependent kinase inhibitor 1A (CDKN1A) and PRMT6-mediated cyclin dependent kinase inhibitor 1b (CDKN1B) to promote colon cancer (Luo et al., 2019). YTH n6-methyladenosine RNA binding protein 2 (YTHDF2) is downstream of epidermal growth factor receptor/src proto-oncogene, non-receptor tyrosine kinase/extracellular signal-regulated kinase (EGFR/SRC/ERK) and plays an important role in the proliferation and invasion of glioma cells. Studies have shown that YTHDF2 mediates the downregulation of liver X receptor-alpha (*LXRA*) mRNA through m6A to influence glioblastoma (GBM) cholesterol homeostasis (Fang et al., 2021). Argininosuccinate synthase 1 (ASS1) in cisplatin-resistant bladder cancer cells is hypermethylated, resulting in greatly downregulated expression, suppressing the apoptotic effects of cisplatin (Yeon et al., 2018).

### Non-Coding Ribonucleic Acids

Recent studies have shown that non-coding RNAs regulate the metabolic remodeling of tumors, including sugar metabolism, lipid metabolism, cholesterol metabolism, and amino acid metabolism (Xu et al., 2021c; Sellitto et al., 2021). However, the mechanism by which long non-coding RNAs regulate tumor metabolism is still unclear. Many studies have explored the relationship between metabolism and non-coding RNAs. Urothelial cancer associated 1 (UCA1) has been shown to increase the activation of HK2 by inhibiting miR203 and to regulate glucose metabolism by increasing glucose uptake and lactic acid production, promoting the proliferation and metastasis of esophageal cancer (Liu H.-E. et al., 2020). CircCDKN2B-AS1, the cyclic structure of the long non-coding RNA CDKN2B-AS1, can combine with IMP U3 small nucleolar ribonucleoprotein 3 (IMP3) to stabilize the transcription of *HK2* and promote aerobic glycolysis and the malignant phenotype in cervical cancer (Zhang Y. et al., 2020). Prostate cancer associated 3 (*PCA3*) targets miR-132–3P and weakens its interaction with SREBP1, leading to lipid metabolism disorders caused by antimony exposure in prostate cancer (Guo et al., 2021) (Figure 1).

**FIGURE 2:**
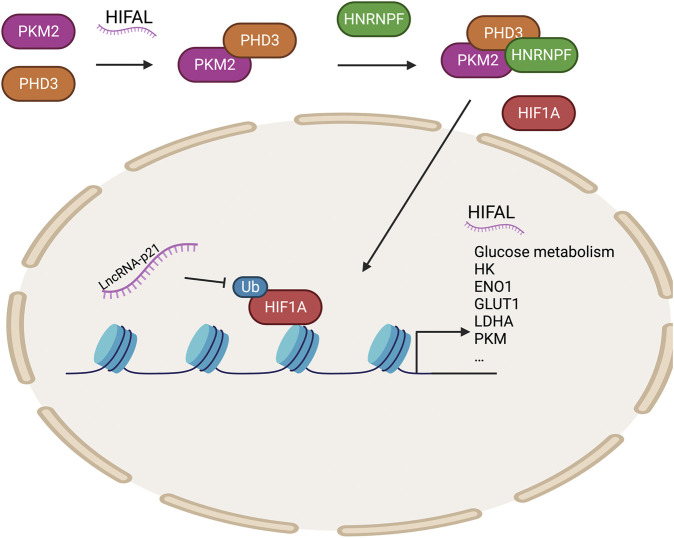
Epigenetic regulation of HIF1A regulates glycolytic enzymes. HIFAL recruits PHD3 to PKM2, and the PKM2/PHD3 complex is then guided by HNRNPF into the nucleus to enhance the transcriptional activation of HIF1α to promote the expression of genes related to glucose metabolism. LncRNA-P21 protects HIF1A stability. PHD3, prolyl hydroxylase domain-containing protein 3; HNRNPF, heterogeneous nuclear ribonucleoprotein F; HIFAL, anti-sense lncRNA of HIF1A; PKM2, pyruvate kinase M2.

HIF1α upregulates the transcription of genes encoding glucose transporters and glycolytic enzymes to regulate tumor glucose metabolism. In recent years, several studies have shown that long non-coding RNAs (lncRNAs) play key roles in regulating the HIF1α pathway (Tan Y. T. et al., 2021). LncRNA-p21 is a hypoxia-reactive lncRNA that can bind HIF1α to inhibit the ubiquitination of HIF1α and then promote glycolysis under hypoxic conditions, contributing to the Warburg effect (Yang et al., 2014). HIFAL (the anti-sense lncRNA of HIF1α) recruits PHD3 to PKM2 to promote the hydroxylation of its proline residues, and the PKM2/PHD3 complex is then guided by heterogeneous nuclear ribonucleoprotein F (hnRNPF) into the nucleus to enhance the transcriptional activation of HIF1α and promote glycolysis (Zheng et al., 2021). Meanwhile, HIF-1α-stabilizing long noncoding RNA (HISLA) inhibits the interaction between prolyl hydroxylase domain-containing protein 2 (PHD2) and HIF1α to prevent the degradation of HIF1α and promote glycolysis, hindering breast cell apoptosis (Chen et al., 2019). Many genes involved in glucose metabolism are transcriptionally activated by HIF1α (Semenza, 2003) (Figure 2).

Recent research on microRNAs has led to increasing evidence on the pivotal role of miRNAs in all stages of tumor development. MiR-9-1 targets HK2 to inhibit glycolysis, reduce the formation of lactic acid, and promote metabolic reprogramming in nasopharyngeal carcinoma cells (Xu Q. L. et al., 2021). Meanwhile, miR-542–3p elevates HK2 to induce glycolysis in GBM (Kim et al., 2021). miRNA-1185-2-3p inhibits Golgi phosphoprotein 3 (GOLPH3L) and affects central carbon metabolism regulated by GOLPH3L (Xu et al., 2021b). MiR-16–5p and 15b-5p coregulate ALDH1A3, which can inhibit the ubiquitination of PKM2 and regulate glycolysis to exert anti-cancer effects (Huang X. et al., 2021). In ovarian cancer, miR-424–5p inhibits mitochondrial elongation factor 2 (MIEF2), which regulates mitochondrial fission, inhibits glucose metabolism from oxidative phosphorylation to glycolysis, and inhibits tumor growth (Zhao et al., 2020). LIX1-like protein (LIX1L) promotes miR-21–3p, inhibits FBP1, reduces lactic acid production, and affects sugar metabolism to inhibit tumor growth (Zou et al., 2021) (Figure 1). MiR-15a-5p inhibits acetate uptake and acyl-CoA synthetase short chain family member 2 (ACSS2) and H4 acetylation in the nucleus under hypoxia, inhibiting fatty acid synthesis in lung cancer cells and further suppressing malignancy in lung cancer (Ni et al., 2020).

### Histone Modifications

Various PTMs act on histones. Most histone modification sites are at the N-terminal end of the nucleosome tail of H3 and H4 histones. Common histone modifications include methylation, acetylation, ubiquitination, SUMOylation, and phosphorylation (Ahringer and Gasser, 2018). However, in recent years, new histone modifications have been discovered, including crotonylation, GlcNAcylation, and citrullination (Blakey and Litt, 2015; Zhang M. et al., 2020).

Different histone modifications are associated with different chromatin states—specifically, the methylation of H3K4 activates transcription, trimethylation at H3K27 is associated with transcription inhibition, acetylation of the histone tail generally promotes transcription, and deacetylation of histones inhibits transcription (Carter and Zhao, 2021). Epigenetic changes control the expression of many metabolic genes, which are important in cancer metabolism (Sun et al., 2021). Increasing evidence shows that histone methylation and metabolic variations in cancer cells are highly correlated (Rausch et al., 2021). Enhancer of zeste 2 polycomb repressive complex 2 subunit (EZH2), a key epigenetic enhancer, suppresses gene transcription by promoting H3K27me3 (Zhang T. et al., 2020). Recently, EZH2 was shown to affect tumor cell metabolism, including carbohydrate metabolism, amino acid metabolism, and lipid metabolism. EZH2 regulates the Warburg effect to promote tumorigenesis and cancer development. EZH2-mediated histone H3 lysine 27 trimethylation mediates the downregulation of LINC00261 to promote glycolysis in pancreatic cancer (Zhai et al., 2021). EZH2 is enriched in the *WNT* promoter region and regulates H3K27me3 to suppress the transcription of *WNTs*, including *WNT-1*, *6*, *10a*, and *10b* genes, which are involved in the process of adipogenesis (Wang et al., 2010). In lung cancer, lysine methyltransferase 2D (KMT2D) is highly expressed and regulates the super enhancers H3K4me1 and H3K27ac of period circadian regulator 2 (PER2; circadian inhibitory factor), affecting the expression of *PER2* and its downstream glycolytic genes (Alam et al., 2020). As a H3K27me2 reader, PHD Finger Protein 20 Like 1(PHF20L1) plays important roles in promoting the Warburg effect via many glycolysis-related genes (GRGs) in breast cancer (Hou et al., 2020). Lysine-specific histone demethylase 1 (LSD1) also plays an important role in the metabolic regulation of hepatocellular carcinoma. LSD1 mediates the methylation of H3K4 to inhibit the expression of PPARgamma coactivator 1alpha (PGC1α), thereby, affecting mitochondrial oxidative respiration (Sakamoto et al., 2015). Meanwhile, the interaction between LSD1 and snail family transcriptional repressor 2 (SNAIL2, also known as SLUG, an important epigenetic regulator of *de novo* adipose tissue) mediates the demethylation of H3K9 and stimulates FASN expression and lipogenesis (Liu Y. et al., 2020; Manuel and Haeusler, 2020). Protein arginine methyltransferase 5 (PRMT5) regulates fat cells by promoting the expression of fatty acid synthase (*FASN*; lipid synthesis gene) by methylating sterol regulatory element binding transcription factor 1 (SREBP1) (Jia et al., 2020).

Acetylation and deacetylation can also affect the transcriptional output of metabolic genes in various working models. EZH2-deficient cells show increased H3K27 acetylation, indicating that acetylation and trimethylation of H3K27 have a repulsive effect in regulating WNT expression (Wang et al., 2010). In contrast, EZH2 can also inhibit the expression of apolipoprotein E (APOE) in adipocytes to promote lipoprotein-dependent lipid accumulation (Yiew et al., 2019). Histone deacetylase 11 (HDAC11) inhibits histone acetylation of serine/threonine kinase 11(*STK11*) promoter to inhibit its transcription, thereby, promoting the glycolysis pathway and leading to tumor stemness (Bi et al., 2021). Sirtuin 6 (SIRT6) blocks the expression of hypoxia inducible factor 1 subunit alpha (*HIF1α*) to regulate glucose homeostasis by regulating the deacetylation of H3K9 (Zhong et al., 2010). Sirtuin 4 (SIRT4) inhibits the expression of sirtuin 1 (SIRT1) by inhibiting glutamine metabolism, and SIRT1 promotes the deacetylation of H4K16 to regulate the stemness of breast cancer cells (Du et al., 2020). Ubiquitously transcribed *TPR* gene on the X chromosome (*UTX*) is enriched in the uncoupling protein 1 (*UCP1*) and PPARG coactivator 1 Alpha (*PGC1α*) promoters and mediates the demethylation of H3K27me3, which interacts with the histone acetyltransferase (HAT) protein CREB binding protein (CBP), leading to the acetylation of H3K27 (H3K27ac). UTX mediates H3K27me3 demethylation and H3K27ac and converts the target gene from a transcriptionally repressed state to a transcriptionally activated state to positively regulate the thermogenesis of brown adipocytes (Zha et al., 2015). Under hypoxia, acetate increases the acetylation of H3K9, H3K27 and H3K56 in the promoter regions of *ACACA* and *FASN* to activate *de novo* lipid synthesis (Gao et al., 2016). E1A binding protein P300/CREB-binding protein (P300/CBP) mediates histone acetylation of H3K18 and H3K27 in hepatocellular carcinoma to regulate the expression of glycolysis-related metabolic enzymes. In addition, the p300 inhibitor B029-2 effectively blocks the metabolic reprogramming of hepatocellular carcinoma (Cai et al., 2021). Histone lactation is also an emerging epigenetic modification of histone lysine residues (Liberti and Locasale, 2020). It depends on tumor protein P53–E1A-binding protein P300 (p53–P300) to promote gene transcription and promote M2-like features in the late phase of M1 macrophage division (Zhang et al., 2019).

Although many studies have focused on the modification of histones, many reports have shown that these histone modification enzymes regulate the modification of non-histone proteins, such as the sirtuin (SIRT) family, the deacetylase histone deacetylase (HDAC) family, the protein arginine n-methyltransferase (PRMT) family (Eom and Kook, 2014; Biggar and Li, 2015; Rodríguez-Paredes and Lyko, 2019; Navas and Carnero, 2021). These proteins, by regulating metabolic enzymes, play an important role in the occurrence and development of tumors.

The SIRT family is an NAD^+^-dependent type III deacetylase. Many members of this family have recently been shown to regulate tumor metabolism through acetylation (Navas and Carnero, 2021). Sirtuin 2 (SIRT2) mediates the deacetylation of isocitrate dehydrogenase (NADP (+)) 1 (IDH1), thereby, promoting IDH1 activity and the production of α-ketoglutarate (α-KG), inhibiting liver metastasis of colon cancer and improving prognosis (Wang et al., 2020). SIRT3 inhibits the acetylation of *IDH2* at K413 and promotes isocitrate dehydrogenase (NADP (+)) 2 (IDH2) activity by promoting IDH2 dimerization to inhibit glycolysis (Zou et al., 2017). Sirtuin 7 (SIRT7) in the liver increases fatty acid uptake and triglyceride synthesis and storage. SIRT7 inhibits the degradation of testicular nuclear receptor 4 (TR4) via the ddb1-and cul4-associated factor 1/damage specific DNA binding protein 1/cullin 4b (DCAF1/DDB1/CUL4B) complex (Yoshizawa et al., 2014) (Figure 1).

The acetylation of K394 (regulated by the deacetylase P300/CBP-associated factor (PCAF) and histone deacetylase 3 (HDAC3) of enolase 2 (ENO2), a key enzyme of glycolysis) inhibits ENO2 activity and glycolysis (Zheng et al., 2020). Gluconeogenesis is an important metabolic process in liver cell homeostasis; however, it is significantly reduced in liver cancer. The tumor suppressor Nur77 interacts with the rate-limiting enzyme PEPCK1 in gluconeogenesis to increase gluconeogenesis and inhibit the Warburg effect in hepatocellular cancer (HCC) to prevent the development of this disease. However, PEPCK1 after SUMOylation becomes unstable and the interaction weakens, inhibiting gluconeogenesis and promoting glycolysis (Bian et al., 2017). In addition, metastasis associated 1 (MTA1) interacts with the transcription factor myc proto-oncogene protein (MYC) to regulate its transcription on the lactate dehydrogenase A (LDHA; an enzyme that catalyzes the production of lactic acid from pyruvate) via its promoter (Guddeti et al., 2019) and MTA1 upregulates HIF1α under hypoxic conditions by stimulating HIFα transcription (Huang W. et al., 2021). Six homeobox 1 (SIX1) is a key transcription factor that regulates glycolysis-related enzymes (GLUT1 and HK2). SIX1 increases the level of O-GlcNAcylation, and its O-GlcNAcylation enhances the stability of SIX1, coordinates glucose metabolism, and promotes the proliferation of HCC (Chu et al., 2020) (Figure 1).

## Epigenetic Regulation by Metabolites and Metabolic Enzymes

Metabolism is an umbrella term for a variety of different orderly chemical reactions that occur in organisms to maintain life and serve cellular demands, including energy generation and protein biosynthesis, thereby, maintaining biological structure and functions and responding to the environment. Metabolism can be regarded as a process of continuous material and energy exchange. Intermediates produced by metabolism often participate in epigenetic regulation by serving as substrates or cofactors for epigenome-modifying enzymes. During environmental disturbances, cellular metabolism transmits regulatory signals to the genome via epigenetic modifications (Reid et al., 2017; Cavalli and Heard, 2019) (Table 1).

**TABLE 1:** Regulation of epigenetic modifications by metabolites and metabolic enzymes.

Metabolites or metabolic enzymes	Epigenetic modifications		Functions in cancer	References
Metabolites				
*Acetyl-CoA*		DNA, RNA and Protein acetylation	Plays a regulatory role in tumorigenesis and development	Gopal and Pizzo (2017)
*NAD* ^*+*^		Protein dacetylation, desuccinylase, demalonylase	Enables tumor progression, development and survival	Hanahan and Weinberg (2011)
*Succinyl CoA*		Protein succinylation	Promotes tumor growth and progression	Li et al. (2020b)
*2-hydroxyglutarate*		Protein hypermethylation	Contributes to poor prognosis	Cavalli and Heard (2019)
*Lactate*		Protein lactylation	Contributes to tumorigenesis and indicates poor prognosis	Yu et al. (2021)
*Palmitic acids*		Protein palmitoylation	Participates in tumorigenesis and cell survival	Yao et al. (2019)
*Farnesyl group*		Protein farnesylation	Contributes to cancer cell growth	Tamanoi et al. (2001)
*Geranylgeranyl group*		Protein geranylgeranylation	Essential for cell proliferation and migration	Dou et al. (2015), Mi et al. (2015); Lin and Yang (2016)
*β-hydroxybutyrate*		Protein β-hydroxybutyrylation	Promotes tumor growth	Liu et al. (2019)
*Glutamine*		Produce a variety of metabolites	Replenishes the TCA cycle for biosynthesis to meet the needs of proliferation	DeBerardinis et al. (2008), Kuhn et al. (2010)
*SAM*		DNA, RNA and Protein methylation	Enhances subcutaneous tumor growth	Dann et al. (2015)
*UDP-GlcNAc*		O-GlcNAcylation	Promotes tumorigenesis	Peng et al. (2017)
Metabolic enzymes				
*ACLY and ACSS1*		Acetylation	Contributes to cancer cell proliferation, migration and invasion	Gopal and Pizzo (2017)
*α-KGDC*		Protein succinylation	Promotes tumor growth and progression	Wang et al. (2017)
*SDH*		Hypersuccinylation; Hypermethylation	SDH loss causes drug resistance and promotes angiogenesis	Isaacs et al. (2005); Selak et al. (2005); MacKenzie et al. (2007); Smestad et al. (2018)
*FH*		Hypersuccinylation; Hypermethylation	FH deficiency results in angiogenesis and EMT	Isaacs et al. (2005); Selak et al. (2005); MacKenzie et al. (2007); Cancer Genome Atlas Research et al. (2016); Sciacovelli et al. (2016)
*IDH1 and IDH2*		Protein hypermethylation	Contributes to poor prognosis	Figueroa et al. (2010); Lu et al. (2012)
*OGT*		*O-GlcNAcylation*	Promotes tumorigenesis	Slawson and Hart (2011); de Queiroz et al. (2014); Yang and Qian (2017)

### Metabolites Serve as Substrates for Epigenetic Modifications

**FIGURE 3:**
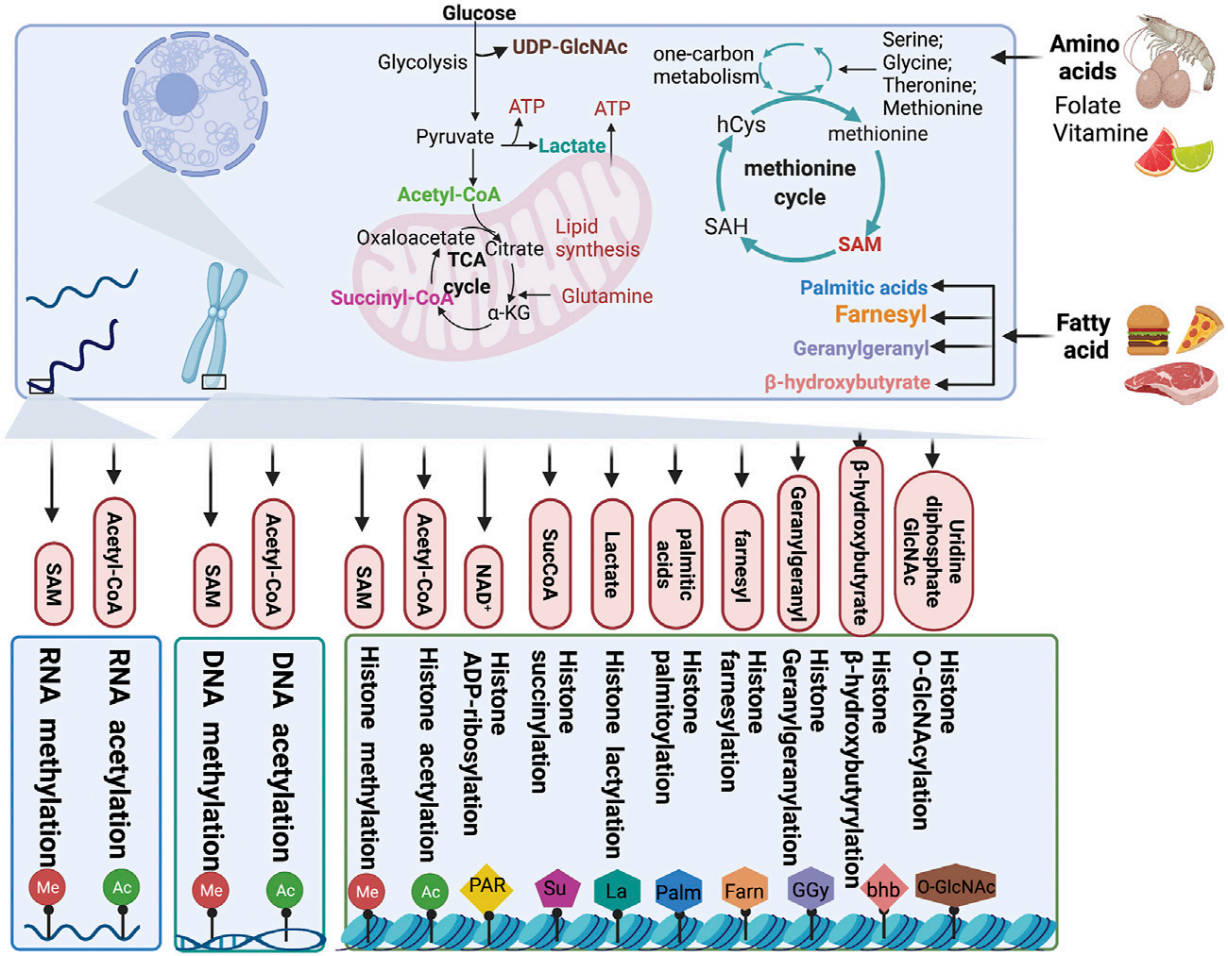
Chromatin modification by metabolites. Metabolites produced from cellular metabolic pathways are used as substrates for DNA, RNA, and chromatin modification. Moreover, the intracellular pools of metabolites can modulate the activity of chromatin-modifying enzymes. TCA, tricarboxylic acid; αKG, α-ketoglutarate; SucCoA, succinyl-CoA; PAR, poly (ADP–ribose); GlcNAc, β-N-acetylglucosamine; SAH, S-adenosylhomocysteine; SAM, S-adenosylmethionine.

As a carrier of epigenetic information, chromatin plays an important role in regulating gene silencing and activation and permits stable inheritance through reversible DNA and histone modification to maintain the biological functions of proteins (Allis and Jenuwein, 2016). In chromatin, DNA and histones are the substrates that are mainly modified. The most widely studied of all modifications are methylation-demethylation, acetylation-deacetylation, phosphorylation, ubiquitination, and ADP-ribosylation. As discussed earlier, changes in the metabolite pool caused by cancer metabolic reprogramming also affect the state of epigenetic modifications. Consequently, metabolic alterations in cancer have an effect on malignant phenotypes of cancer cells by manipulating epigenetic modifications (Figure3).

### Glucose Metabolism

Glycolysis involves the breakdown of glucose or glycogen into pyruvate and nicotinamide adenine dinucleotide (NADH), accompanied by the production of a small amount of ATP. Under anoxic conditions, pyruvate can accept hydrogen from triose phosphate and gets reduced to lactate under the catalytic action of lactate dehydrogenase. Under aerobic conditions, pyruvate can be further oxidized and decomposed to form acetyl-CoA and enter the TCA cycle. Intermediates and metabolites, such as acetyl-CoA, α-KG, succinate, fumarate and citrate, produced from glucose metabolism, have diverse effects on epigenetic modifications.

#### Acetyl-CoA

Acetyl-CoA is a core metabolite and a substrate for further metabolism and acetylation. A variety of metabolic processes in cells participate in the formation of the acetyl-CoA pool. Glucose metabolism intermediates, such as acetate, citrate, and pyruvate, under the catalytic action of acetyl-CoA synthetase short-chain family member (ACSS), ATP citrate lyase (ACLY), and the pyruvate dehydrogenase complex (PDC), respectively, produce acetyl-CoA. In addition to glucose metabolism, the metabolism of amino acids and fatty acids also generate acetyl-CoA (Zaidi et al., 2012; Nagaraj et al., 2017; Sivanand et al., 2018). Acetylation occurs widely in histones and some non-histone proteins and plays a regulatory role in tumorigenesis and development.

Histone acetylation is regulated by the intracellular acetyl-CoA concentration. Acetyl-CoA serves as an acetyl group donor in acetylation reactions. This process is catalyzed by HATs (Lee et al., 2014). Histone acetylation is determined by the ratio of acetyl-CoA/coenzyme A and is generally deregulated in cancer. In human gliomas and prostate tumors, Histone acetylation marks are significantly regulated by pAkt (Ser473) levels, which modulates the metabolic enzyme ATP-citrate lyase, a key determinant of acetyl-CoA metabolism (Lee et al., 2014). Tumor cell surface GRP78 of activated α2-macroglobulin signals regulate tumor cell proliferation by inducing and activating ACLY and ACSS1 expression to generate acetyl-CoA (Gopal and Pizzo, 2017). Acetyl-CoA also serves as nonhistone acetylation modification substrate to produce effects on the development of tumors. PHD finger-like domain-containing protein 5A (PHF5A) is acetylated at lysine 29 by p300 to promote cancer cell capacity for stress resistance and consequently, contributes to colon carcinogenesis. PHF5A K29 hyperacetylation induces the alternative splicing of *KDM3A* mRNA, which enhances its stability and promotes its expression (Wang H.-Y. et al., 2019; Wang Z. et al., 2019). Under low oxygen states or hypoxia, lysine acetyltransferase and CBP rely on acetate-dependent acetyl CoA synthetase 2 (ACSS2) to acetylate HIF-2, which contributes to cancer cell proliferation, migration, and invasion (Chen et al., 2015).

#### Nicotinamide Adenine Dinucleotide (NAD^+^)

The metabolite NAD^+^ serves as a catalytic cofactor for sirtuins, class III HDACs, and is essential for the deacetylation of lysine residues of sirtuins. NAD^+^ homeostasis is related to many diseases, including neurodegeneration, diabetes, and cancer. The ratio of NAD^+^/NADH regulates sirtuins activity, which is found to be higher in cancer cells than in noncancerous cells (Houtkooper et al., 2012; Moreira et al., 2016; Katsyuba et al., 2020). Under genotoxic stress or nutrient restriction conditions, upregulation of the NAD^+^ biosynthetic enzyme NAMPT protects cells against death via the deacetylation activity of SIRT3 and SIRT4 (Yang et al., 2007). In cancer, NAD^+^ is intended to reprogram metabolism to enable tumor progression, development, and survival (Hanahan and Weinberg, 2011). As an NAD^+^-consuming enzyme, SIRT1 acts as a tumor promoter and is upregulated in several human cancers. In HCC, the inhibition of NAD^+^ metabolism causes DNA damage in the early stages of tumorigenesis because of the inactivation of NAD^+^-consuming enzymes such as SIRT1. Genomic stability can be improved by NAD^+^ supplementation to prevent tumorigenesis (Tummala et al., 2014). Apart from deacetylase activity, sirtuins have multiple NAD^+^-dependent catalytic functions, such as desuccinylase, demalonylase, demyristoylase, depalmitoylase, and/or mono-ADP-ribosyltransferase activities. In ovarian cancers, nicotinamide mononucleotide adenylyl transferase 2 (NMNAT2), which mediates the synthesis of NAD^+^ is highly upregulated. Knockdown of NMNAT2 significantly decreases NAD^+^ in cytoplasm but increases NAD^+^ in the nucleus and consequently supports the catalytic activity of the mono (ADP-ribosyl) transferase (MART) PARP-16, resulting in ribosome mono-ADP-ribosylation (MARylation). There is a significant positive correlation between NMNAT-2 and MARylation levels in the samples of ovarian cancer patients, and a high level of MARylation will lead to poor prognosis with respect to progression free survival (Challa et al., 2021). In addition, mitochondrial dysfunction also affects the level of acetylation. Mitochondrial dysfunction causes a decreased NAD^+^/NADH ratio and increased reactive oxygen species (ROS), resulting in senescence (Wiley and Campisi, 2016). STAT3 deficiency induces senescence, mitochondrial dysfunction, and a lower NAD^+^/NADH ratio (Igelmann et al., 2021). The dynamic changes of NAD^+^/NADH ratio also has an effect on nonhistone deacetylation of lysine residues of sirtuins. In breast cancer, NAMPT causes p53 deacetylation and SIRT1 activation by increasing the NAD^+^ pool (Behrouzfar et al., 2017).

#### α-Ketoglutarate, Succinyl-CoA, Fumarate

Intermediates of the TCA cycle, α-KG, succinyl-CoA, fumarate, and citrate, regulate epigenetic modifications through their concentration in the metabolite pool. α-KG is a key cofactor for jumonji-domain-containing histone demethylases (JHDMs), which synergistically catalyze histone demethylation (Klose et al., 2006). In Acute Myeloid Leukemia (AML) stem cells, the α-KG level is restricted by the branched-chain amino acid transaminase 1 (BCAT1), which is overexpressed in leukemia stem cells and transfers α-amino groups from BCAAs to α-KG, resulting in α-KG instability to maintain leukemia stem-cell function (Raffel et al., 2017). The α-KG dehydrogenase complex (α-KGDC) is the hub enzyme of various metabolic pathways involved in mitochondrial function and is a modulator of α-KG (Vatrinet et al., 2017). Succinyl-CoA is a substrate for succinylation (Zhang et al., 2011). Succinylation, as a post-translational modification of proteins, can convert the side chain of cationic lysine residues into anionic ones and then affect the structure and function of proteins (Smestad et al., 2018). The increase of succinylation has different functions in the progression of a variety of tumors. α-KGDC binds to the promoter regions of lysine acetyltransferase 2A (*KAT2A*, also known as *GCN5*), and KAT2A also acts as a succinyltransferase and succinylates histone H3 on lysine 79, which facilitates histone succinylation and tumor cell proliferation (Wang et al., 2017). Lysine-222 succinylation is increased in gastric cancer, and LDHA lysine-222 succinylation, catalyzed by CPT1A via the stabilization of LDHA, promotes GC invasion and proliferation (Li X. et al., 2020). In pancreatic ductal adenocarcinoma, succinyl-CoA synthetase ADP-forming subunit β (SUCLA2) phosphorylation at S79 leads to dissociation from kidney-type glutaminase (GLS). Thus, the concentration of succinyl coenzyme A is increased and upregulates the succinylation of GLS at site K311. GLS K311 succinylation enhances the oligomerization, activity, and glutaminolysis to improve the concentration of nicotinamide adenine dinucleotide phosphate (NADPH) and glutathione, thereby, counteracting oxidative stress and promoting tumor growth (Tong et al., 2021).

Loss-of-function mutations in the TCA cycle enzymes fumarate hydratase (FH) and succinate dehydrogenase (SDH) have been identified as driver mutations in cancer and mediators of epigenetic reprogramming (Pollard et al., 2003). In an SDH-loss cell model, the accumulation of succinate and succinyl-CoA results in global lysine hypersuccinylation, which modulates genome-wide transcription and hinders DNA repair ability and drug resistance (Smestad et al., 2018). In paraganglioma, SDH deficiency causes succinate accumulation and establishes a hypermethylation phenotype resulting from epigenetic silencing through the inhibition of 2-OG-dependent histone and DNA demethylases. In addition to SDH, inactivating FH mutations can also lead to this phenomenon (Letouze et al., 2013). The hypermethylation phenotype caused by SDH-inactivating mutations also exists in gastrointestinal stromal tumors (Killian et al., 2013). In renal cancer, the inactivating mutations of SDH or FH deficiency results in the subsequent accumulation of succinate or fumarate, respectively, thus, the inhibition of hypoxia-inducible factor (HIF) prolyl hydroxylases (HPH), which protect the stabilization of HIF and promote angiogenesis by increasing α-KG intake can alleviate this situation (Isaacs et al., 2005; Selak et al., 2005; MacKenzie et al., 2007). Moreover, germline mutations of FH cause an aggressive and metastatic form of type 2 papillary renal-cell carcinoma, which is linked to a widespread DNA hypermethylation pattern (Cancer Genome Atlas Research et al., 2016). Loss of FH triggers epigenetic suppression of miR-200 and epithelial-to-mesenchymal transition by inhibiting Tet-mediated demethylation (Sciacovelli et al., 2016).

#### 2-Hydroxyglutarate

Gain-of-function isocitrate dehydrogenase (IDH) mutations produce an oncometabolite, 2-hydroxyglutarate (2-HG), specifically the D enantiomer (D-2HG), and affect clinical therapy and prognosis (Dang et al., 2010; Cancer Genome Atlas Research et al., 2015). IDH1 and IDH2 mutations result in global hypermethylation and specific hypermethylation in AML by inhibiting demethylases such as KDM4C (Figueroa et al., 2010; Lu et al., 2012). 2-HG-mediated inhibition of the H3K9 demethylase KDM4C is induced during adipocyte differentiation (Lu et al., 2012). In addition to affecting the function of methyltransferase, 2-HG might also perturb the overall architecture of the genome and contribute to cancer and prognosis by affecting methyltransferase activity (Cavalli and Heard, 2019). Interestingly, kidney tumors present an accumulation of the L enantiomer of 2HG (L-2HG), because of the low expression of L-2HG dehydrogenase (L2HGDH). Similar to the function of D-2HG, L-2HG inhibits ten-eleven translocation (TET) as well, which converts 5-methylcytosine (5mC) to 5-hydroxymethylcytosine (5hmC). L-2HG is significantly increased in tumors compared with their expression in normal kidney, and L2HGDH expression inhibits proliferation and colony formation in RCC cells (Shim et al., 2014). In response to hypoxia, L-2HG is selectively produced to increase histone methylation levels, especially histone 3 lysine 9 (H3K9me3) (Intlekofer et al., 2015).

#### Lactate

Lactate, a metabolic by-product of pyruvate metabolism in cancer cells, is a substrate for histone lactylation. The lactylation of histone lysine residues (Kla) driven by lactate directly stimulates gene transcription from chromatin, linking metabolism and gene regulation. The production of intracellular lactic acid is the main determinant of lactylation modification. Therefore, the balance between glycolysis and mitochondrial metabolism can regulate lactylation modification (Izzo and Wellen, 2019; Zhang et al., 2019; Liberti and Locasale, 2020). Histone lactylation is elevated in ocular melanoma and is indicative of poor prognosis. Histone lactylation also contributes to tumorigenesis by facilitating *YTHDF2* expression, which acts as an m6A reader to recognize and mediate the degradation of m6A-modified Period Circadian Regulator 1 *PER1* and *P53* mRNAs to accelerate tumorigenesis (Yu et al., 2021).

### Lipid Metabolism

Lipid signal transduction, especially the phosphatidylinositol 3 kinase (PI3K) pathway, is one of the most common signal transduction systems in cancer cells. Lipophilic groups are widespread modifiers and the following five types of lipids can be covalently attached to proteins: fatty acids, isoprenoids, sterols, phospholipids, and glycosylphosphatidylinositol anchors (Resh, 2013). In addition to providing a substrate for rare acylation, fatty acid metabolism also produces acetyl-COA, as a substrate for energy metabolism and epigenetic modifications (Edmunds et al., 2014).

#### Palmitic Acid

Palmitoylation is a type of fatty acylation in which long-chain fatty acids (usually 16-carbon-long palmitic acid) are covalently modified to protein cysteine residues by thioester bonds. Palmitoylation plays a vital role in many biological processes (Zhang et al., 2018). In several human cancers, signal transducer and activator of transcription 3 (STAT3) is palmitoylated by zinc finger DHHC-Type palmitoyltransferase 19 (ZDHHC19), a palmitoyl acyltransferase, at the SRC homology 2 (SH2) domain, which promotes its dimerization and transcriptional activation. Under cytokine stimulation, the association between ZDHHC19 and STAT3 is promoted, which directly activates STAT3 by enhancing its palmitoylation with fatty acids (Niu et al., 2019). Palmitoylation mediated by the palmitoyltransferase zinc finger DHHC-Type palmitoyltransferase 3 (ZDHHC3) in the cytoplasmic domain of PD-L1 blocks its ubiquitination to stabilize PD-L1, thereby, suppressing PD-L1 degradation by lysosomes and PD-L1-mediated immune evasion in cancer (Yao et al., 2019). In addition to ZDHHC3, ZDHHC9 has been identified as a palmitoyltransferase for PD-L1 in breast cancer (Yang et al., 2019). In p53-mutant glioma, zinc finger DHHC-Type palmitoyltransferase 5 (ZDHHC5) is transcriptionally upregulated and mediates EZH2 palmitoylation, which affects methyltransferase activity of EZH2 and causing trimethylation of histone 3 at lysine 27 (H3K27me3 level) decreased, and then contributes to malignancy and tumor progression (Chen X. et al., 2017). MC1R palmitoylation mediated by the protein-acyl transferase zinc finger DHHC-Type palmitoyltransferase (ZDHHC13) activates MC1R signaling, which triggers increased pigmentation, ultraviolet-B-induced G1-like cell cycle arrest, and the control of senescence and melanomagenesis. Thus, MC1R palmitoylation protects against melanomagenesis (Chen S. et al., 2017). The c-Met β-chain is palmitoylated at the cysteine site, which enhances its stability and release from the Golgi for transport to the plasma membrane, making it a novel therapeutic target for c-Met-driven cancers (Coleman et al., 2016). Palmitoyltransferases are upregulated in GBM and induce marked palmitoylation of proteins that participate in cell survival control and cell cycle regulation in GBM (Chen et al., 2020).

#### Farnesyl Group

Protein farnesylation is a lipid PTM essential for the cancer-causing activity of proteins, such as gtpase Ras (Sebti, 2005). Farnesyltransferase inhibitors inhibit cancer cell growth and regulate cell cycle changes (Tamanoi et al., 2001).

#### Geranylgeranyl Group

GGylation is an essential modification affecting cell survival in many types of cancer. In breast cancer, the Hippo pathway mediates GGylation-dependent cell proliferation and migration (Dou et al., 2015; Mi et al., 2015; Lin and Yang, 2016). In gastric cancer, GGylation promotes proliferation, migration, and invasion of gastric cancer cells through the YAP signaling pathway. Inhibition of GGylation by the mevalonate pathway inhibitor atorvastatin and the geranylgeranyltransferase I inhibitor GGTI-298 can impair cell proliferation and migration (Wei et al., 2020).

#### β-Hydroxybutyrate

Acetyl CoA produced by lipolysis can not only enter the TCA cycle for oxidation but also result in the synthesis of ketone bodies in the liver. β-Hydroxybutyrate (β-HB) is a ketone body produced by fatty acid hydrolysis. Elevated β-HB levels lead to a new type of histone mar, lysine β-hydroxybutyrylation (Kbhb) (Xie et al., 2016). β-HB is an endogenous and specific inhibitor of class I HDACs (Shimazu et al., 2013). In HCT116 cells, β-HB-mediated p53 Kbhb at K120, K319, and K370 sites results in lower levels of p53 acetylation and consequently, decreased activity of p53, leading to weakened tumor-suppressive function (Liu et al., 2019). MTA2 can transcriptionally regulate BDH1-mediated histone β-HB modification through the R-loop structure in synergy with HDAC2/CHD4 and promote the proliferation of hepatoma stem cells (Zhang et al., 2021).

### Amino Acid and One-Carbon Metabolism

One-carbon metabolism plays a crucial role in integrating metabolites from amino acids, glucose, and vitamins and participating in a variety of biosynthetic processes, including the biosynthesis of lipids, nucleotides, and proteins. Hyperactivation of the one-carbon metabolism pathway drives oncogenesis and forges links between cellular epigenetic status and metabolism (Locasale, 2013). Serine metabolism is also an important determinant of S-adenosylmethionine (SAM) levels. Serine is a single carbon donor of the folate cycle and is used for methionine regeneration and SAM synthesis (Locasale, 2013).

#### Glutamine

Glutamine is the most abundant amino acid in the intracellular metabolite pool. After entering a cell, in the mitochondria, glutamine is transformed into glutamate, the precursor of the TCA cycle intermediate α-KG. Cells rely on glutamine to replenish the TCA cycle. Anaplerosis allows cancer cells to use the TCA cycle for biosynthesis to meet the needs of proliferation, and glutamine as an indispensable nutrient therein (DeBerardinis et al., 2008; Kuhn et al., 2010).

#### SAM

SAM, a major methyl donor, is synthesized from the methionine cycle and serves as a substrate for methylation to maintain the epigenetic status, including histone methylation catalyzed by histone methyltransferases, DNA methylation mediated by DNA methyltransferase (DNMT), RNA methylation (Teperino et al., 2010; Varier and Timmers, 2011; Anderson et al., 2012; Locasale, 2013), and PTMs such as the methylation of lysine and arginine residues of nonhistone proteins (Chen et al., 2011; Yang and Bedford, 2013). Amino-acid transporters are often required for tumor cell import of essential amino acids. The amino acid transporters LAT1 and LAT4 are significantly upregulated in tumors to facilitate the uptake of methionine for cell proliferation and differentiation in cancer cells (Haase et al., 2007). In lung cancer, cells that highly express LAT1 show increased abundance of SAM, improving the activity of the histone methyltransferase EZH2 and consequently, increasing H3K27me3 levels to enhance subcutaneous tumor growth (Dann et al., 2015). The levels of SAM are controlled by multiple metabolic pathways and intracellular metabolism, as well as environmental inputs, such as nutrient availability, which also elevate SAM concentrations.

#### Uridine Diphosphate N-Acetylglucoseamine (UDP-GlcNAc)

The hexosamine biosynthetic pathway produces nucleotide sugars that can be used as substrates for glycosylation. UDP-GlcNAc is a metabolite of the hexosamine biosynthetic pathway, which integrates glucose, amino acid, fatty acid, and nucleotide metabolism. O-GlcNAc transferase (OGT) adds O-GlcNAc groups from the donor substrate UDP-GlcNAc to proteins for O-GlcNAcylation (Yang and Qian, 2017; Li et al., 2018). The methylcytosine dioxygenases TET1, TET2, and TET3 can also become O-GlcNAcylated by OGT through protein–protein interactions (Hardiville and Hart, 2016; Yang and Qian, 2017). Previous studies have shown that O-GlcNAc glycosylation is abnormally upregulated in many tumors and plays an essential role in the proliferation of malignant cancer cells (Slawson and Hart, 2011; de Queiroz et al., 2014; Yang and Qian, 2017). Phosphoglycerate kinase 1 (PGK1) O-GlcNAcylation levels are significantly upregulated in colorectal cancer; O-GlcNAcylation activates PGK1 activity to upregulate the TCA cycle and results in lactate production. The O-GlcNAcylation of PGK1 at T255 increases colon cancer cell proliferation. Furthermore, PGK1 T255 O-GlcNAcylation contributes to tumorigenesis by promoting glycolysis and upregulating the TCA cycle (Nie et al., 2020). Yes-associated protein (YAP) is O-GlcNAcylated at serine 109 by OGT, and the O-GlcNAcylated YAP disrupts the interaction with the upstream kinase large tumor suppressor kinases (LATS) 1. Consequently, O-GlcNAcylation activates the transcriptional activity of YAP by preventing the phosphorylation mediated by LATS1. Thus, glucose-induced YAP O-GlcNAcylation and activation promote tumorigenesis (Peng et al., 2017). OGT O-GlcNAcylates SRPK2 at a nuclear localization signal and triggers SRPK2 binding to importin α, which results in the import of SRPK2 into the nucleus and the phosphorylation of serine/arginine-rich proteins, promoting the splicing of lipogenic pre-mRNAs. O-GlcNAc promotes tumor growth by enhancing the intracellular localization of SRPK2 and regulating *de novo* lipid synthesis in tumor cells at the post-transcriptional level (Tan W. et al., 2021).

### Epigenetic Regulation by Gut Microbiota-Mediated Changes in the Metabolic Pool

**FIGURE 4:**
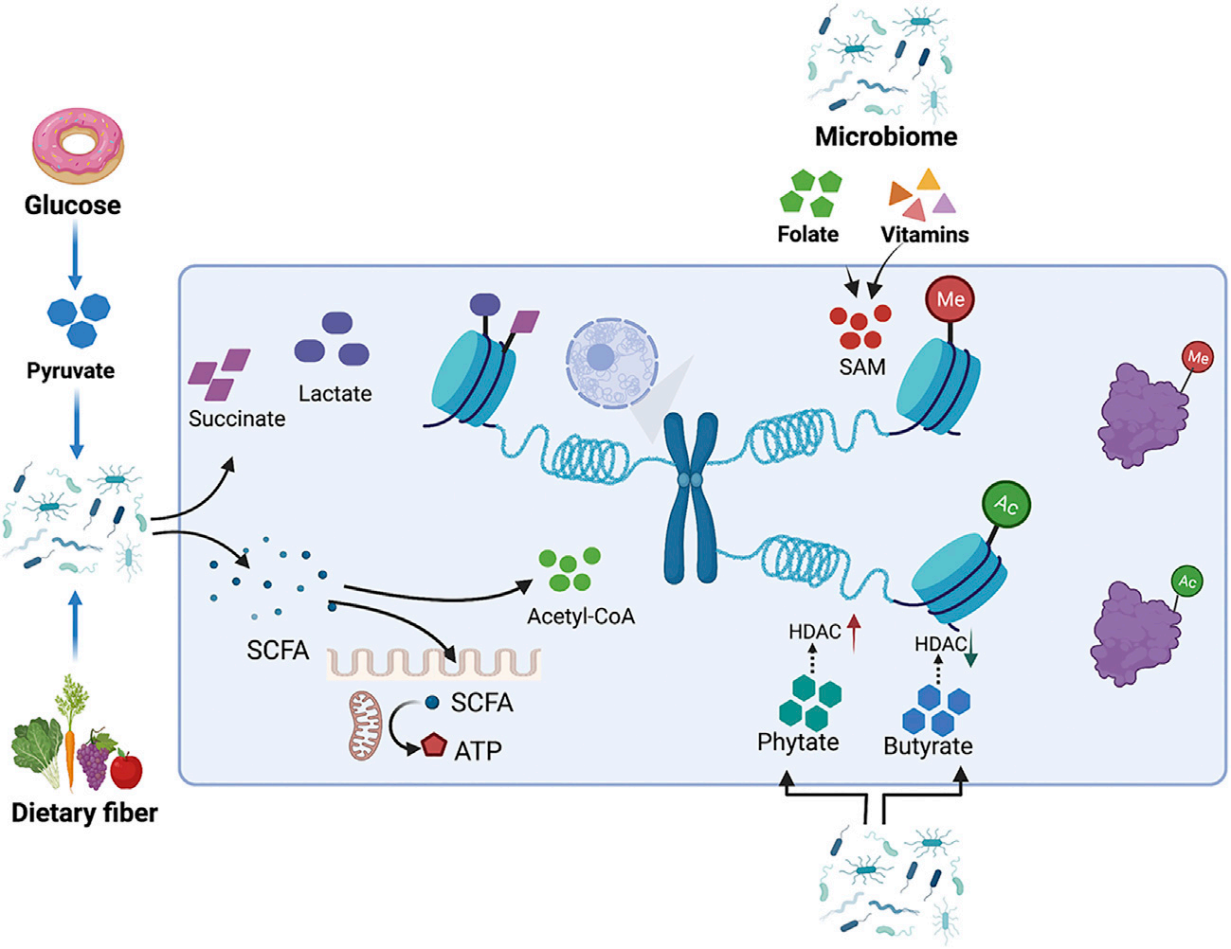
Metabolites produced by the intestinal microbiota influence host metabolism by modulating the metabolite pool. The gut microbiome regulates histone methylation and acetylation by modulating the intracellular pools of metabolites, such as SAM and acetyl-CoA. Metabolites from the gut microbiome, like phytate and butyrate, also cause changes in histone acetylation by affecting the enzyme activity of HDAC. Pyruvate can either be catabolized into succinate, lactate, or acetyl-CoA. SCFAs can provide acyl-CoA as a donor for histone acylation, while also directly inhibiting the activity of HDACs.

The biochemical reaction networks of metabolism can be manipulated by several factors including diet, nutrition, gut microbiota, and chemical exposure. These environmental factors regulate chromatin methylation and acetylation by modifying the intracellular pool of metabolites. There is increasing evidence that the gut microbiome is closely related to the risk, development, and progression of cancer. Metabolites produced by gut microbiota influence host metabolism via the modulation of metabolites, including the lipopolysaccharide endotoxin, bile acids, trimethylamine N-oxide, and short-chain fatty acids (SCFAs) (Schroeder and Backhed, 2016). Microbiota-derived metabolites represent stimuli that regulate epigenetic modifying enzymes and are involved in intestinal inflammation and carcinogenesis (Figure 4).

#### Bile Acids

Bile acids are produced in the liver and metabolized by enzymes produced by intestinal bacteria. Intestinal bacteria are very important for maintaining a healthy intestinal microbiota, balancing lipid and glucose metabolism, insulin sensitivity, and natural immunity. Bile acids have a wide range of gene-mediating effects, including bile acid metabolism, glucose and lipid metabolism, energy expenditure, intestinal motility and bacterial growth, inflammation, liver regeneration, and hepatocarcinogenesis (de Aguiar Vallim et al., 2013; Wahlstrom et al., 2016). The epigenetic regulation of cofactors senses changes in bile acid metabolism, regulates PTMs of histones, and causes chromatin remodeling to regulate gene transcription and maintain the balance of bile acids. Bile acids activate Farnesoid X receptor (FXR) and change the interaction between FXR and transcriptional cofactors, resulting in altered PTMs of FXR and histones to effectively modulate the expression of target genes (Kemper, 2011). An abnormal concentration of bile acids is believed to promote colorectal cancer (Fearon and Vogelstein, 1990).

#### Butyrate

Butyrate is a microbiota-derived SCFA that has been confirmed to be an inhibitor of HDAC *in vitro* and *in vivo*. Exposure to butyrate causes the accumulation of acetylated histones (Candido et al., 1978; Wu S.-e. et al., 2020). In intestinal epithelia, histone H3K18 results in abundant crotonylation modification, especially in small intestine crypts and the colon; the class I histone deacetylases HDAC1, HDAC2, and HDAC3 can catalyze histone decrotonylation. As an HDAC inhibitor, gut microbiota-derived butyrate affects histone decrotonylation and is linked to gene regulation (Fellows et al., 2018). Clostridial clusters, *Anaerostipes*, and *Eubacterium* cause histone acylation modification through butyrate, thus, improving intestinal development and the immune balance (Wu J. et al., 2020). Butyrate increases histone methylation in the promoter region of *NF-κB1*, downregulating its expression (Liu et al., 2016; Yang and Yu, 2018).

#### Phytate

Phytate digestion and inositol phosphates produced by phytate metabolism induce HDAC activity in intestinal epithelial cells. Dietary phytate metabolism mediated by microbiota-dependent mechanisms improves recovery from intestinal damage. Moreover, microbiota-derived phytate digestion is significantly decreased in the intestinal contents of ulcerative colitis patients relative to that in inflammatory bowel disease patients. In addition, phytate-treated mice exhibit improved survival compared to vehicle-treated mice with dextran sodium sulfate-induced colitis (Wu S.-e. et al., 2020).

The human gut microbiota also catabolizes the conversion of pyruvate into succinate, lactate, or acetyl-CoA to further generate energy (Oliphant and Allen-Vercoe, 2019); this process is accompanied by changes in the metabolite pool. Dietary fiber, including non-starch polysaccharides, resistant starch, and oligosaccharides, are very rich and diverse in our diet and facilitate gut microbial and consequently beneficial metabolite diversity (Riva et al., 2019). The gut microbiota break cellulose into SCFAs as a metabolic intermediate; SCFAs inhibit HDACs and serve as energy substrates. SCFAs also affect histone crotonylation and acetylation levels (Kasubuchi et al., 2015; Koh et al., 2016; Zhao et al., 2016; Fellows et al., 2018; Sabari et al., 2018). SCFAs induce epithelial anti-inflammatory IL-10 receptor alpha subunit (*IL-10RA*) mRNA and antimicrobial peptides by inhibiting HDACs (Campbell et al., 2012; Zheng et al., 2017). *Lactobacillus* and *Bifidobacterium* synthesize folate to increase DNA methylation and m6A mRNA in the intestine to ensure normal development of the intestine (Wu J. et al., 2020). In addition to folate, vitamins B2, B6, and B1 from intestinal microbiota can also lead to the synthesis of SAM as a methyl group donor to methylate DNA, RNA, and histones (Wu J. et al., 2020). Microbial metabolism also provides TCA cycle intermediates, such as α-KG, succinate, and fumarate, which serve as substitutes for epigenetic modifications (Wu J. et al., 2020). The gut microbiota also indirectly regulates the activity of epigenetically modified genes by regulating gene expression. According to a recent report, enterotoxigenic *Bacteroides fragilis* stimulation promotes PHF5A-mediated RNA alternative splicing of *KAT2A* via the downregulation of miR-149–3p, promoting intestinal inflammation and malignancy. This process depends on METTL14-mediated m6A methylation (Cao et al., 2021).

## Clinical Trials

Recent studies have shown that tumor metabolism and epigenetic regulation are closely linked and important for maintaining cell growth and regulating cancer metastasis. Therefore, inhibitors of rate-limiting enzymes in key metabolic pathways used by cancer cells are actively being investigated (Erez and DeBerardinis, 2015). Inhibiting such key enzymes has potential for cancer treatment, but they would also have an effect on normal cells; therefore, inhibiting rate-limiting enzymes of metabolic pathways is often toxic (Rodríguez-Enríquez et al., 2014).

In 2017, the FDA approved the first cancer metabolism drug enasidenib, an IDH2 inhibitor, for which the main indication is relapsed and refractory AML (Mullard, 2017). IDH2 is the key rate-limiting enzyme in the TCA cycle and catalyzes the conversion of isocitrate to α-KG. Cancer cells show a functional mutation in *IDH2* (Yao et al., 2021). Enasidenib, an IDH2 inhibitor, is undergoing phase 1b/2 clinical trials in multiple countries. The current research results show that compared to that with azacitidine treatment alone, the overall drug response rate of enasidenib combined with azacitidine is improved, and the combination has good drug tolerance, thereby, improving the prognosis of patients with IDH2 AML (NCT02677922) (Table 2) (DiNardo et al., 2021). In addition, clinical studies have also shown that the drug responsiveness and tolerability are good in patients with IDH2-mutant myelodysplastic syndrome (NCT01915498) (Table 2) (Stein et al., 2020).

**TABLE 2:** Clinical trials of IDH, HDAC, SAM cycle and others.

Category	Drugs	NCT number	Title	Condition	Status	Phase
IDH inhibitors	Enasidenib	NCT02677922	A Safety and Efficacy Study of Oral AG-120 Plus Subcutaneous Azacitidine and Oral AG-221 Plus Subcutaneous Azacitidine in Subjects with Newly Diagnosed Acute Myeloid Leukemia (AML)	Active, not recruiting	Leukemia, Myeloid, Acute	Phase 1/Phase 2
	Enasidenib	NCT01915498	Phase 1/2 Study of Enasidenib (AG-221) in Adults with Advanced Hematologic Malignancies with an Isocitrate Dehydrogenase Isoform 2 (IDH2) Mutation	Active, not recruiting	Hematologic Neoplasms	Phase 1/Phase 2
	AG881	NCT02481154	Study of Orally Administered AG-881 in Patients with Advanced Solid Tumors, Including Gliomas, with an IDH1 and/or IDH2 Mutation	Active, not recruiting	Glioma	Phase 1
HDAC inhibitors	Romidepsin	NCT00426764	A Trial of Romidepsin for Progressive or Relapsed Peripheral T-cell Lymphoma	Completed	Peripheral T-cell Lymphoma	Phase 2
	Romidepsin	NCT00106431	A Single Agent Phase II Study of Romidepsin (Depsipeptide, FK228) in the Treatment of Cutaneous T-cell Lymphoma (CTCL)	Completed	Cutaneous T-cell Lymphoma	Phase 2
	Vorinostat	NCT01266031	Phase I/II Adaptive Randomized Trial of Bevacizumab Versus Bevacizumab Plus Vorinostat in Adults with Recurrent Glioblastoma	Completed	Recurrent Glioblastoma	Phase 1/Phase 2
SAM cycle inhibitors	Ethylornithine	NCT01483144	Trial of Eflornithine Plus Sulindac in Patients with Familial Adenomatous Polyposis (FAP)	Completed	Familial Adenomatous Polyposis	Phase 3
	Ethylornithine	NCT00033371	Celecoxib With or Without Eflornithine in Preventing Colorectal Cancer in Patients with Familial Adenomatous Polyposis	Completed	Colorectal Cancer, Familial Adenomatous Polyposis	Phase 2
	Ethylornithine	NCT01059071	Safety Study for Refractory or Relapsed Neuroblastoma with DFMO Alone and in Combination with Etoposide	Completed	Neuroblastoma	Phase 1
Others	Physical activity and dietary change	NCT00811824	Effects of Physical Activity and Dietary Change in Minority Breast Cancer Survivors	Completed	Breast Cancer	Phase 2
	Vitamin C	NCT02877277	Epigenetics, Vitamin C and Abnormal Hematopoiesis - Pilot Study	Completed	Myelodysplastic Syndrome, Acute Myeloid Leukemia	Not Applicable

*IDH* mutations result in the conversion of α-KG to 2-hydroxyglutarate, which competitively inhibits α-KG-dependent enzymes as the two small molecules, 2-HG and α-KG, are similar in structure (Pirozzi and Yan, 2021). These enzymes include histone demethylases (Jumonji domain-containing protein/lysine demethylase family, JMJD/KDM family) and DNA/RNA methylation-modification-related enzymes (Tet family) (White et al., 2020; Waitkus and Yan, 2021). This causes epigenetic dysregulation and cell differentiation arrest, a key factor in tumor cell proliferation after mutations (White et al., 2020). In addition, in 2018, the mutant IDH1 inhibitor ivosidenib was used to treat relapsed or refractory AML. The IDH1 and IDH2 dual inhibitor AG-881 has also been shown to cross the blood-brain barrier. Recently, the results of a phase I clinical trial (NCT02481154) (Table 2) of AG-881 for low-grade gliomas demonstrated that it is safe and can shrink tumors in many non-enhanced glioma patients (Mellinghoff et al., 2021).

Histone deacetylase is a key target for tumor therapy. Therefore, many studies have explored compound inhibitors of histone deacetylase to find new anti-tumor strategies. HDAC inhibitor romidepsin (also called FK228) affects iron metabolism, mediates increased intracellular iron accumulation, decreases the expression of export-type ferroprotein, and increases reactive oxygen species (ROS) production, thereby, mediating iron death. This result has a guiding role for the combined treatment strategy using HDAC inhibitors and iron-targeted chemotherapy (Oliveira et al., 2021). The results of the second-phase clinical study show that FK228 can effectively control T-cell lymphoma, with safety and reliability (NCT00426764, NCT00106431) (Table 2) (Foss et al., 2014; Foss et al., 2016). The HDAC inhibitor BAS-2 inhibits HDAC6, affects the *ENO1* gene and *LDHA* related to glucose metabolism, and ultimately affects glycolysis in breast cancer (Dowling et al., 2021). Studies have shown that 1A12 can inhibit the acetylation level of histone H3, and it can also affect the glucose metabolism level in preclinical test subjects (Chan et al., 2014). HDAC inhibitors (panobinostat, vorinostat, and romidepsin) reduce glycolysis in a c-MYC-dependent manner, triggering the metabolic reprogramming of glioblastoma (Nguyen et al., 2020). Currently, the HDAC inhibitors vorinostat, romidepsin, and panobinostat have been approved by the FDA for the treatment of clinical lymphoma and multiple myeloma (Cappellacci et al., 2020). However, current research shows that vorinostat does not work well in glioma (NCT01266031) (Table 2) (Puduvalli et al., 2020).

SAM is a methyl donor for DNA and histone methylation (Haws et al., 2020). Metformin has been found to affect the epigenetic metabolic regulation of tumors in recent years. It reduces SAH (a potent inhibitor of all SAM-dependent methylation reactions) and promotes the accumulation of SAM. This leads to an increase in the overall DNA methylation level of metastatic cancer cells (Cuyàs et al., 2018). One study found that ethylornithine has chemopreventative and therapeutic effects on colon cancer, reducing folate-dependent metabolites such as SAM, the thymidine pool, and other intermediate products of related pathways (Witherspoon et al., 2013). On one hand, ethylornithine has an important effect in delaying the progression of familial polyposis (NCT01483144, NCT00033371) (Table 2) (Burke et al., 2020), while on the other, studies have shown that it has the potential to prevent the recurrence of high-risk neuroblastoma (NCT01059071) (Table 2) (Saulnier Sholler et al., 2015).

In addition to drugs, lifestyle changes can affect epigenetic changes. A healthy lifestyle is associated with high levels of DNA methylation, whereas overall tissue and blood DNA hypomethylation are associated with an increased risk of cancer. The results of clinical trials (NCT00811824) (Table 2) show that lifestyle changes (diet and weight loss) can effectively change DNA methylation (Delgado-Cruzata et al., 2015). A high-fat diet can induce DNA methylation changes in the whole genome, which was found to involve metabolic disease-related genes and cancer-related genes (Jacobsen et al., 2012). Previous studies have shown that patients with malignant hematological tumors often lack vitamin C. Vitamin C can enhance the activity of TET protein in tumor cells, increase the level of 5hmC, decrease the level of 5mC in cells, and increase sensitivity to anticancer drugs. A clinical study in Denmark (NCT02877277) (Table 2) showed that vitamin C supplementation in patients with myeloma increases the 5hmC/5mC ratio of mononuclear myeloid cells, enhancing the clinical efficacy of DNMT inhibitors (Gillberg et al., 2019).

## Conclusion and Perspectives

In this review, we elucidated how the crosstalk between epigenetic regulation and metabolic reprogramming changes during the process of tumorigenesis and development. Metabolic reprogramming in tumors changes the epigenetic landscape of tumor cells. Metabolites can serve as substrates for epigenetic modifications, and some metabolic enzymes can also affect chromatin modification. Therefore, we suggest that the metabolism-epigenome axis must be considered while approaching cancer biomarker studies.

Additionally, the role of metabolism synergy and epigenetic modifications in the physiology and pathology of the gut microbiota and corresponding effect on the host is proposed to be valuable and have prospective applications in cancer research. On the one hand, the metabolism of the gut microbiota and the products provide substrates for epigenetic modifications, whereas on the other hand, they directly affect the activity of epigenetic modification enzymes, resulting in epigenetic modification changes. Lifestyle-related factors, such as exercise and nutrition, are very important factors that affect human health through the gut microbiota, and the emerging role of gut microbial diversity in epigenetics emphasizes the link between lifestyle choices and cancer.

Although great progress has been made in understanding the epigenetic–metabolism axis in the past few decades, the internal mechanism of this heritable change in tumorigenesis and development and its application to tumor prevention and treatment remain elusive. Therefore, the mechanisms of metabolically-regulated epigenomic landscape responses in tumorigenesis and development need to be investigated, and further potential therapeutic applications in this regard must be studied.
